# MFN2 mediates ER-mitochondrial coupling during ER stress through specialized stable contact sites

**DOI:** 10.3389/fcell.2022.918691

**Published:** 2022-09-08

**Authors:** Benjamin Gottschalk, Zhanat Koshenov, Olaf A. Bachkoenig, René Rost, Roland Malli, Wolfgang F. Graier

**Affiliations:** ^1^ Molecular Biology and Biochemistry, Gottfried Schatz Research Center, Medical University of Graz, Graz, Austria; ^2^ BioTechMed Graz, Graz, Austria

**Keywords:** mitochondria, ER stress, mitochondrial Ca^2+^, mitofusin 2, mitochondria-associated membranes (MAM)

## Abstract

Endoplasmic reticulum (ER) functions critically depend on a suitable ATP supply to fuel ER chaperons and protein trafficking. A disruption of the ability of the ER to traffic and fold proteins leads to ER stress and the unfolded protein response (UPR). Using structured illumination super-resolution microscopy, we revealed increased stability and lifetime of mitochondrial associated ER membranes (MAM) during ER stress. The consequent increase of basal mitochondrial Ca^2+^ leads to increased TCA cycle activity and enhanced mitochondrial membrane potential, OXPHOS, and ATP generation during ER stress. Subsequently, OXPHOS derived ATP trafficking towards the ER was increased. We found that the increased lifetime and stability of MAMs during ER stress depended on the mitochondrial fusion protein Mitofusin2 (MFN2). Knockdown of MFN2 blunted mitochondrial Ca^2+^ effect during ER stress, switched mitochondrial F_1_F_O_-ATPase activity into reverse mode, and strongly reduced the ATP supply for the ER during ER stress. These findings suggest a critical role of MFN2-dependent MAM stability and lifetime during ER stress to compensate UPR by strengthening ER ATP supply by the mitochondria.

## Introduction

Contact sites between mitochondria and the endoplasmic reticulum (ER) are important structures facilitating Ca^2+^ signaling (Patergnani et al., 2011) and lipid transport (Vance, 2014) within both organelles. Especially Ca^2+^ transport across the inter-organelle cleft was extensively studied. The discovery of ER and mitochondrial contact sites (Rizzuto et al., 1998) led to the discovery that mitochondrial-associated ER membranes (MAMs) serve as Ca^2+^ hotspots (Giacomello et al., 2010) facilitating the Ca^2+^ flow from the ER to mitochondria. The composition of MAMs spans a wide field of proteins and protein complexes. Besides the PTPIP51-VAPB (Protein tyrosine phosphatase-interacting protein 51/Vesicle-associated membrane protein-associated protein B) complex, the IP_3_R/Grp75/VDAC-complex (Kania et al., 2017; Valladares et al., 2018) is considered as most important protein assembly linking ER and mitochondrial. The inositol 1,4,5-triphosphate receptor (IP_3_R) releases Ca^2+^ from the ER and the voltage-dependent anion channel (VDAC1) is responsible for Ca^2+^ transfer through the outer mitochondrial membrane (OMM). The two proteins are tethered *via* the glucose-regulated protein 75 (Grp75) (Honrath et al., 2017; Xu et al., 2018), enabling efficient and fast transition of Ca^2+^ from the ER to the mitochondria.

Mitofusion2 (MFN2) is another protein that is important for ER-mitochondria tethering (de Brito and Scorrano, 2008). Moreover, MFN2 mediates mitochondrial fusion (Rojo et al., 2002; Filadi et al., 2018) and participates in the control of respiration, autophagy, and mitochondrial movement regulation (Filadi et al., 2018). Notably, MFN2 knockout was shown to increases the distances between ER and mitochondria (Naon et al., 2016; Casellas-Díaz et al., 2021). However, others reported that knockout and knockdown of MFN2 lead to decrease ER-mitochondria distances (Filadi et al., 2015). This points to an role of MFN2 as “distance holder” within the two organelles while achieving the optimal distance between ER and mitochondria of 15–20 nm (Lim et al., 2021). Too short (<7 nm) or to big distances between the ER and mitochondria can impede the Ca^2+^ transfer (Csordás et al., 2010). Mitochondrial Ca^2+^ uptake *via* MAMs leads to the activation of the tricarboxylic acid (TCA) cycle and stimulates ATP syntheses (Hurst et al., 2017). MAMs were associated lately with the transport of ATP into the ER *via* AXER (ATP/ADP exchanger in the ER membrane) (Klein et al., 2018) and the [Ca^2+^]_cyto_ inhibited [ATP]_ER_ uptake CaATiER (Ca^2+^-Antagonized Transport into ER) (Yong et al., 2019).

ER stress is characterized generally by the activation of UPR due to the accumulation of misfolded proteins. During ER-stress, ATP is utilized by chaperones (Goloubinoff et al., 1989), valosin-containing protein (VSP) during ER-associated degradation (ERAD) (Kobayashi et al., 2002; Okamoto et al., 2010), and the proteasome for degradation of misfolded proteins (Ogura and Tanaka, 2003). Accordingly, an increase in [ATP]_ER_ during ER stress is essential to support the organelle in its action against stress, i.e., ER stress response (Yong et al., 2019). Nevertheless, an analysis on the role of MAMs and mitochondrial to ER transport of ATP for ER stress response is still missing. We studied the ER-mitochondrial interaction dynamics during the early phase of ER stress induced by tunicamycin. We used structured illumination super-resolution microscopy (SIM) to track single MAMs over time and correlate these dynamics with live-cell imaging of [ATP]_ER_, [ATP]_mito_ and [Ca^2+^]_mito_ using genetically encoded fluorescent biosensors. Further, membrane potential (Ψ_mito_), oxygen consumption rate (OCR), and [Ca^2+^]_cyto_ measurements were complemented to investigate the contribution of ER-mitochondrial contact sides to ER stress response. Moreover, while it is still under debate whether MFN2 serves as an ER-mitochondria tether (Naon et al., 2016), spacer (Filadi et al., 2015) or both, increased MFN2 expression occurs under ER-stress (Ngoh et al., 2012) and MFN2 knockout severely increases UPR upon ER-stress induction by tunicamycin (Muñoz et al., 2013). Therefore, we investigated the influence of MFN2 knockdown on MAM dynamics and correlated ER-mitochondrial contacts to [ATP]_ER_, [ATP]_mito_, and [Ca^2+^]_mito_.

## Material and methods

### Structured illumination microscopy

Single and dual camera SIM imaging. The SIM-setup used is composed of a 405, 488, 515, 532, and a 561 nm excitation laser introduced at the back focal plane inside the SIM-box with a multimodal optical fiber. For super-resolution, a CFI SR Apochromat TIRF 100x-oil (NA 1.49) objective was mounted on a Nikon-Structured Illumination Microscopy (N-SIM^®^, Nikon, Austria) System with standard wide field and SIM filter sets and equipped with two Andor iXon3^®^ EMCCD cameras mounted to a Two Camera Imaging Adapter (Nikon Austria, Vienna, Austria). At the bottom port a third CCD-camera (CoolSNAP HQ2, Photometrics, Tucson, United States) is mounted for wide field imaging. For calibration and reconstruction of SIM images the Nikon software (NIS-Elements, Nikon, Austria) was used. Reconstruction was permanently performed with the same robust setting to avoid artefact generation and ensures reproducibility with a small loss of resolution of 10% compared to most sensitive and rigorous reconstruction settings. Microscopy setup adjustments were done as described elsewhere (Gottschalk et al., 2019).

### Mitochondrial morphology and Mito-ER co-localization

Cells expressing ERAT4.03 NA (NGFI, Graz, Austria) were stained with 100 nM TMRM in EH-loading buffer [135 mM NaCl, 5 mM KCl, 2 mM CaCl2, 1 mM MgCl2, 10 mM HEPES, 2.6 mM NaHCO3, 440 μM KH2PO4, 340 μM Na2HPO4, 10 mM D-glucose, 0.1% MEM vitamins (Gibco), 0.2% MEM amino acids (Gibco), 1% penicillin-streptomycin (100 U/ml), 1% streptomycin (100 μg/ml), and 1.25 μg/ml amphotericin B, pH adjusted to 7.4] (Gottschalk et al., 2019) for 20 min and imaged. Morphology parameters were measured automatically *via* a custom-made ImageJ macro using the following procedure. An additional background subtraction based on the rolling ball method was introduced to enhance contrast for later analysis. A global auto Otsu threshold using a stack histogram as well as a local auto Otsu threshold (radius of 640 nm) based on a single slice histogram were applied and merged. ERAT4.03 NA staining was Otsu thresholded, dilated, filled, and subsequently and used as a mask for TMRM stained mitochondria to exclude not transfected cells.

Binarized mitochondria were segmented using the ImageJ “Analyse particles” function. Mitochondrial area, aspect ratio, and form factor were analyzed using ferrets diameter and perimeter as previously described (Gottschalk et al., 2019).

To analyze the co-localization between ER and mitochondria, TMRM and ERAT4.03 NA were determined on a single cell level using ImageJ and plugin coloc2. The Pearson’s coefficient was calculated as previously described (Tawfik et al., 2022).

### Measurement of ER-mitochondrial interaction in single MAMs

Cells expressing ERAT4.03 NA (ER-channel) (NGFI, Graz, Austria; www.ngfi.eu) were stained with 100 nM TMRM (Mito-channel) in EH-loading buffer for 20 min and imaged over time for 10 min. ER- and Mito-channel were background subtracted using the “rolling ball” background subtracter implemented in ImageJ. Pixel wise multiplication of ER- and Mito-channel was performed, followed by a pixel wise calculation to the potency of two. This was done to increase the contrast of ER and mitochondrial overlapping regions. Next, we used the Mosaic Particle Tracker (Sbalzarini and Koumoutsakos, 2005) out of the Mosaic suit plugin collection for ImageJ to identify and track single interactions between the ER and mitochondria. The Mosaic Particle Tracker Plugin was used to detect particles dependent on the contrast and size through an image stack and connects the particles afterwards to create the traces visible in the Supplementary Videos. Out of the x and y coordinates the plugin automatically calculates duration, velocity and a manifold of other results which can be extracted as txt-file.

### Transfection procedures

HeLa or EAhy926 cells were grown under standard culture conditions until 50% confluence was reached, transfected in DMEM (without FCS and antibiotics) with 1.5 µg/well plasmids or 100 µM siRNA (MFN2 siRNA: 5′-CGG CAA GAC CGA CUG AAA U dTdT-3′; Control siRNA 5′-UUC UCC GAA CGU GUC ACG UTT-3′) using 2.5 µg/well TransFast™ transfection reagent (Promega, Madison, WI, United States). Next day, the medium was replaced with DMEM containing 10% FCS and 1% penicillin/streptomycin and kept for further 24 h prior experiments. Alternatively, plasmid transfection was done by using PolyJet™ *In Vitro* DNA Transfection Reagent (SL100688, SignaGen^®^ Laboratories, Frederick, MD, United States).

### Measurements of [Ca^2+^]_mito_, [ATP]_mito_, [ATP]_ER_ and mitochondrial membrane potential

Dynamic changes in [Ca^2+^]_mito_ were analyzed in HeLa cells expressing the organelle-targeted FRET-based Ca^2+^ sensor 4mtD3cpv as previously described (Tawfik et al., 2022). [ATP]_ER_ was measured in cells expressing the ER targeted genetically encoded ATP sensor ERAT 4.01. Cells were stimulated with the IP3-generating agonist ATP (Sigma Aldrich, Vienna, Austria). Basal levels in [ATP]_mito_ were measured in HeLa cells expressing the organelle-targeted FRET-based ATP sensor mtAT1.03 (Depaoli et al., 2018). To determine mitochondrial membrane potential, cells were incubated in 20 nM of the fluorescent indicator tetramethylrhodamine (TMRM) (Invitrogen™ T668; Vienna, Austria) in EH-loading buffer for 20 min at room temperature, as described previously (Depaoli et al., 2018). A full disruption of the mitochondrial membrane potential by application of carbonyl cyanide-p-trifluoromethoxyphenylhydrazone (FCCP) (Abcam, Cambridge, United Kingdom) was used to normalize the TMRM_mito_/TMRM_nuc_ ratio.

Measurements were performed on an inverted wide-field microscope (Observer.A1, Carl Zeiss GmbH, Vienna, Austria) as described previously (Depaoli et al., 2018; Koshenov et al., 2021) that was equipped with an programmable gravity-based perfusion system (NGFI). In short, cells were imaged with a ×40 oil immersion objective (Plan Apochromat 1,3 NA Oil DIC (UV) VISIR, Carl Zeiss GmbH, Vienna, Austria) and a standard CFP/YFP filter cube. Emission was collected with a 505dcxr beam-splitter on two sides of the camera (CCD camera, Coolsnap Dyno, Photometrics, Tucson, AZ, United States). 4mtD3cpv, ERAT4.01, and mtAT1.03 were excited with a wavelength of 440 nm (440AF21, Omega Optical, Brattleboro, VT, United States), and emission was captured at 480 and 535 nm (480AF30 and 535AF26, Omega Optical, Brattleboro, VT, United States). TMRM was excited at 550 nm, and the emission was collected at 600 nm. A region of interest containing the mitochondrial TMRM fluorescence (TMRM_mito_) and the cytosolic TMRM (generally the area of the nucleolus) fluorescence (TMRM_nuc_) was selected, and a ratio TMRM_mito_/TMRM_nuc_ was calculated over time. The data were recorded with the NIS-Elements AR software (Nikon, Vienna, Austria) and analyzed using GraphPad Prism (GraphPad Software, San Diego, CA). Measurements were background corrected using a background region of interest and corrected for bleaching using an exponential decay fit of the basal fluorescence extrapolated to the whole measurement. Results are shown as Δmax (TMRMmito/TMRMnuc) between basal level and maximal FCCP induced depolarization.

### Measurements of [Ca^2+^]_cyto_

To measure [Ca^2+^]_cyto_, cells were incubated with the fluorescent cytosolic Ca^2+^ indicator Fura-2 acetoxy-methyl-ester (Fura-2AM) (TEFLabs, Austin, TX) for 30 min in EH-loading buffer, as described previously (Tawfik et al., 2022). An NGFI AnglerFish C-Y7G imager equipped with a Nikon CFI Super Fluor 40 x oil NA 1.3 objective (NGFI) equipped with the NGFI PS9D perfusion system (NGFI, Graz, Austria) was used. Fura-2AM was illuminated with 340 and 380 nm, and emission was captured at 515 nm (495dcxru; Omega Optical, Brattleboro, VT, United States). The results of the measurements were recorded with live-acquisition software v2.0.0.12 (Till Photonics) and analyzed using GraphPad Prism (GraphPad Software 9.3.0, San Diego, CA). The measurement was background-subtracted using a background ROI and corrected for bleaching using an exponential decay fit. Results are shown as the ratio of F_380_/F_340_.

### Live-cell imaging experiments

For live-cell imaging experiments, cells were seeded on 30 mm glass coverslips in 6-well plates and accordingly transfected with 1.5 μg of plasmid DNA and 100 μM of the respective siRNA. Before the measurement, cells were kept in EH-loading buffer at room temperature. During the measurement, the cells were continuously perfused with a Ca^2+^-containing buffer, which consisted of 145 mM NaCl, 5 mM KCl, 2 mM CaCl_2_, 1 mM MgCl2, 10 mM D-glucose, and 10 mM HEPES, pH adjusted to 7.4, using a perfusion system (PS9D, NGFI, Graz, Austria; www.ngfi.eu).

### mRNA isolation and qRT-PCR

Total cellular RNA was isolated from HeLa cells using the PEQLAB total RNA isolation kit (Peqlab, Erlangen, Germany), followed by reverse transcription to cDNA, performed in a thermal cycler (Peqlab) using the high-capacity cDNA reverse transcription kit (Applied Biosystems, Foster City, United States). The qRT-PCR reaction was set up with the GoTag^®^ qPCR Master Mix (Promega, Mannheim, Germany) together with gene-specific primers (Invitrogen, Vienna, Austria). Experiments were performed on a LightCycler 480 (Roche Diagnostics, Vienna, Austria). Relative expression of specific genes was normalized to human GAPDH, as a reference gene. Primer sequences are as follows: MFN1 for: 5′-GCT​CTT​CTC​TCG​ATG​CAA​CT -3′, MFN2 rev: 5′- TGG​CGC​TCT​CCT​GGA​TGT​AG -3′, GAPDH (QuantiTect^®^ Primer Assay Hs_GAPDH, Qiagen, Hilden, Germany).

### Measurement of mitochondrial respiration

35,000 HeLa cells were plated on XF96 polystyrene cell culture microplates (Seahorse^®^, Agilent, CA, United States) a day before the experiment. Thirty minutes before the measurement, the cell medium was changed to XF assay medium supplemented with 1 mM sodium pyruvate, 2 mM glutamine and 5.5 mM D-glucose and incubated in non-CO_2_ 37°C incubator. The XF96 extracellular flux analyzer was used to measure oxygen consumption rate (OCR). OCR (pmol O_2_/min) values were normalized to the protein content. Protein concentration of each well was determined with the PierceTM BCA Protein Assay Kit (Thermo Scientific, Rockford, IL, United States).

### Western blot

Western blots were performed according to standard protocols. Briefly, cell lysis was conducted with RIPA buffer (+1% Triton-X100) (Bio-Rad formulation) supplemented with protease inhibitor cocktail (1:50; Sigma Aldrich, Vienna, Austria). Samples were frozen in liquid nitrogen and thawed for three times and incubated for 30 min on ice. Protein amounts were measured with Pierce BCA Protein Assay Kit (ThermoScientific, United States). 40 µg protein samples were loaded on a 12.5% SDS-PAGE gel together with PageRuler™ Plus Prestained Protein Ladder (Fisher Scientific, Vienna Austria). Blots were blocked (5% milk) and antibodies diluted in 5% milk in TBS-Tween. The following antibodies were used: Mitofusin-2 (D2D10, 1:1,000, Cell Signaling Technology, MA, United States), and β-Actin (D6A8, 1:1,000, Cell Signaling Technology). HRP labeled goat-anti-rabbit (sc-2054, 1:5,000, Santa Cruz Biotechnologies) was used as secondary antibody. For visualization, the SuperSignal West Pico PLUS kit (Fisher Scientific) was used and detection was conducted on a ChemiDoc System (Bio-Rad Laboratories, Vienna, Austria).

### Statistical analysis and reproducibility

Each exact *n* value and the number of independent experiments is indicated in each figure legend. Statistical analysis was performed using the GraphPad Prism software version 9.3.1 (GraphPad Software, San Diego, CA, United States) or Microsoft Excel (Microsoft Office 2016). Analysis of variance (ANOVA) with Tukey post hoc test and unpaired two sided *t*-test were used for evaluation of the statistical significance. *p* < 0.05 was defined to be significant. At least three experiments on three different days were performed for each experimental setup.

## Results

### ER stress changes dynamics of mitochondrial and ER interaction

**FIGURE 1:**
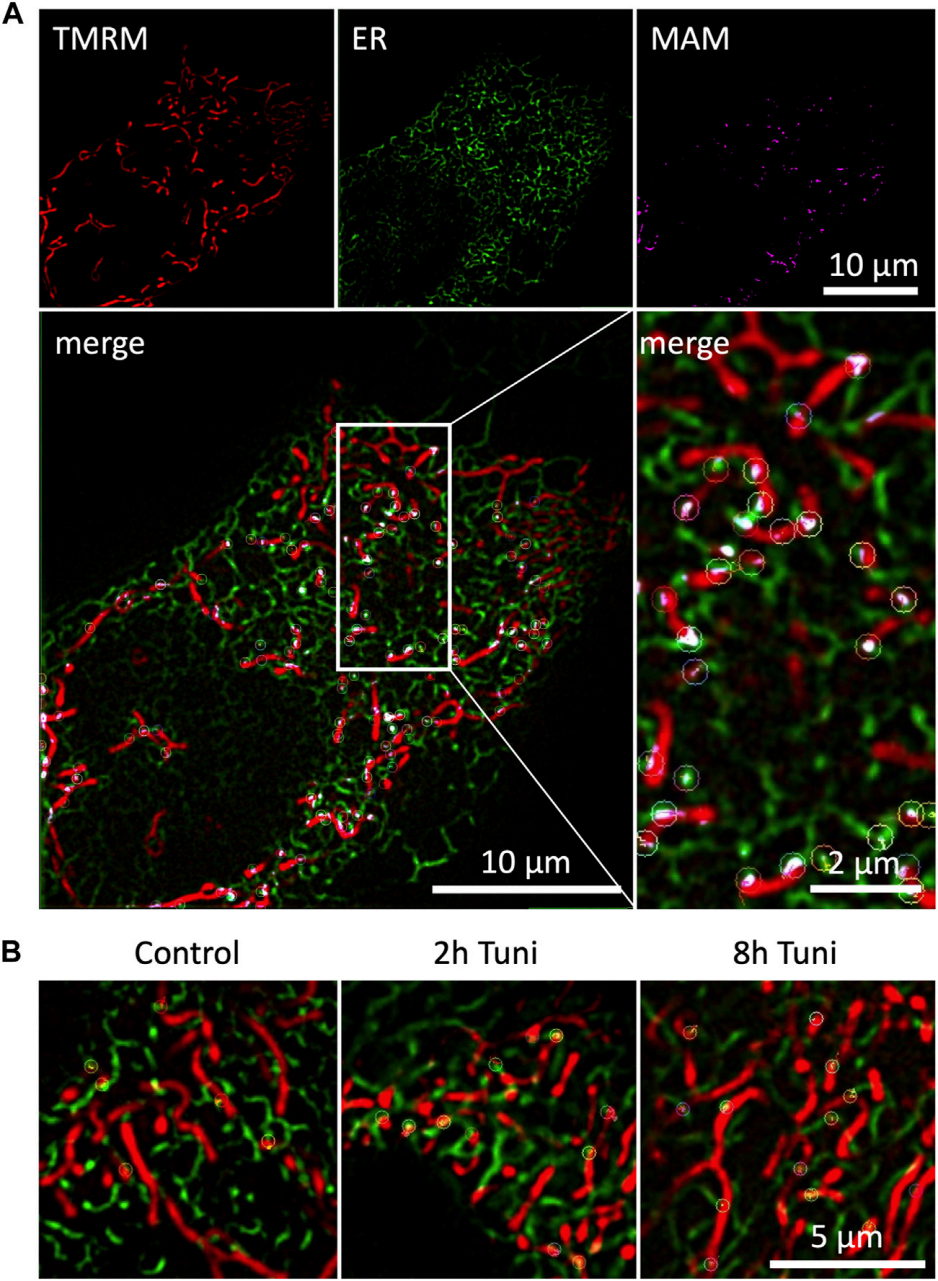
Duration of dynamic ER-mitochondria interactions are prolonged during ER-stress. **(A)** HeLa cells expressing the ER marker ERAT4.01 NA were labeled with 100 nM TMRM and imaged with SIM microscopy. Pixel-wise multiplication of mitochondrial and ER fluorescence followed by a signal amplification resulted in a representation of mitochondrial associated ER membranes (MAM), which were tracked over time and highlighted with color coded circular markers. **(B)** HeLa cells expressing the ER marker ERAT4.01 NA were labeled with 100 nM TMRM and treated with 0.6 µM tunicamycin for two or 8 h or the respective DMSO control. Tracked MAMs are highlighted with color coded circular markers.

**FIGURE 2:**
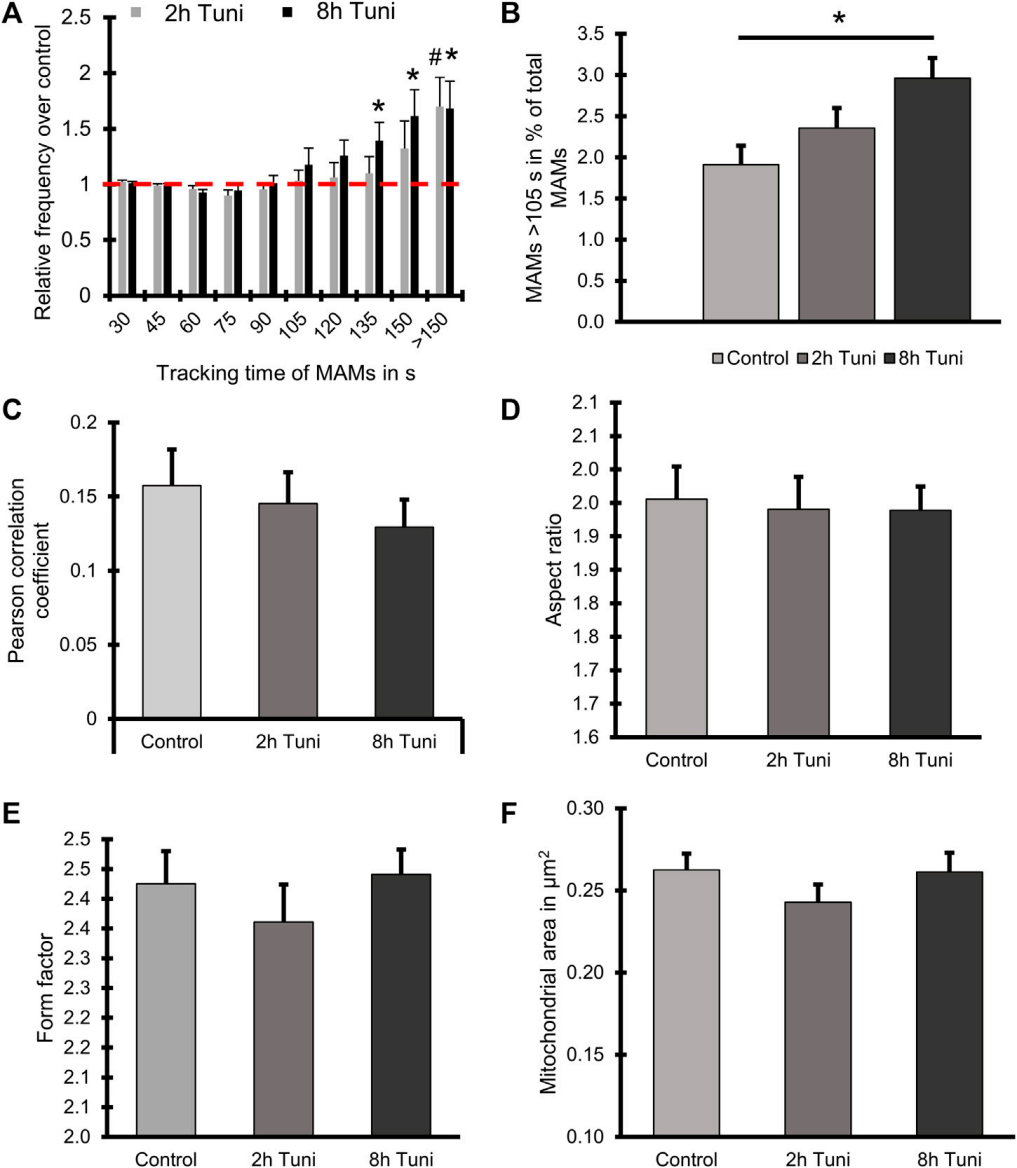
Tunicamycin treatment leads to prolonged ER-mitochondrial contacts but does not influence ER-mitochondrial proximity or mitochondrial morphology. **(A)** HeLa cells expressing the ER marker ERAT4.01 NA were labeled with 100 nM TMRM and treated with 0.6 µM tunicamycin for two or 8 h or the respective DMSO control. The relative frequency of MAMs is plotted over the tracking time of single MAMs. **(B)** A threshold of 105 s tracking time was set to define long lasting MAMs and the percentage of MAMs included in that group was plotted as a bar graph (MEAN ± SEM) for cells treated with tunicamycin for two or 8 h or the respective DMSO control. **(C)** Bar graphs (MEAN ± SEM) shows Pearson’s correlation coefficient of control HeLa or HeLa cells treated with tunicamycin for 2 h or 8 h. **(D)** Bar graphs (MEAN ± SEM) shows mitochondrial aspect ratio of control HeLa or HeLa cells treated with tunicamycin for 2 h or 8 h. **(E)** Bar graphs (MEAN ± SEM) shows mitochondrial form factor of control HeLa or HeLa cells treated with tunicamycin for 2 h or 8 h. **(F)** Bar graphs (MEAN ± SEM) shows mitochondrial size of control HeLa or HeLa cells treated with tunicamycin for 2 h or 8 h *n* = 4/32 (days/cells) #*p* < 0.05 vs. 2 h Tuni respective control and *p < 0.05 8 h Tuni vs. respective control conditions carried out with one-way ANOVA with Tukey corrected posthoc test.

ER-mitochondrial interaction under ER stress is a widely studied topic that is predominantly investigated by high-resolution microscopy, co-localization studies (Bravo et al., 2011) and electron microscopy (Csordás et al., 2006; Bravo et al., 2011). We are aware that structured illumination microscopy has an inferior optical resolution compared to electron microscopy. Nevertheless, there are several advantages of live cell super resolution microscopy compared to electron microscopy. First and most importantly stands the fact that we are able to monitor the cells without fixation and accompanying artefacts. SIM also has a better resolution compared to conventional confocal microscopy without intense illumination and light induced cell toxicity. Besides, the approach has the big advantage of fast preparation and sample rate. Nevertheless, the dynamic assembly and disassembly of inter-organellar contact sites, called MAMs, under ER stress remains elusive, while representing an important qualitative characteristic of MAMs. Therefore, we investigated the dynamics of MAMs in response to the induction of ER stress by tunicamycin. We used ERAT4.01 NA expressing (Vishnu et al., 2014) (ER-targeted fluorescent protein) and TMRM (mitochondrial specific staining) to visualize MAMs. The overlap of the ER and mitochondria stainings was used to track single MAMs (Figure 1A). Induction of ER stress led to more stable MAMs, which could be tracked for longer time (Figure 1B; Supplementary Videos S1–S3). A detailed analysis showed that fractions of MAMs, which exist longer than 105 s, are enriched in cells treated with tunicamycin for 8 h (Figure 2A, Supplementary Figure S1). We set a threshold for the tracking duration of >105 s and observed that stable MAMs were significantly increased by approximately 50% during ER stress (Figure 2B). Additionally, we measured the MAM tracking velocity which was reduced after 8 h tunicamycin treatment (Supplementary Figure S2). The overall number of MAMs measured by the Pearson correlation coefficient of TMRM and ERAT4.01 remained unchanged. (Figure 2C). The mitochondrial morphology, revealed as aspect ratio and form factor, or the average mitochondrial size, were not affected by the early ER-stress (Figures 2D–F). We further evaluated EAhy926 cells regarding MAM duration, ER and mitochondrial colocalization and mitochondrial morphology upon treatment with tunicamycin. Likewise to HeLa cells tunicamycin treatment lead to longer MAM tracking times but unchanged ER-mitochondrial colocalization and morphology (Supplementary Figure S3).

### Tunicamycin increases in basal [Ca^2+^]_mito_ and [Ca^2+^]_cyto_ oscillations

**FIGURE 3:**
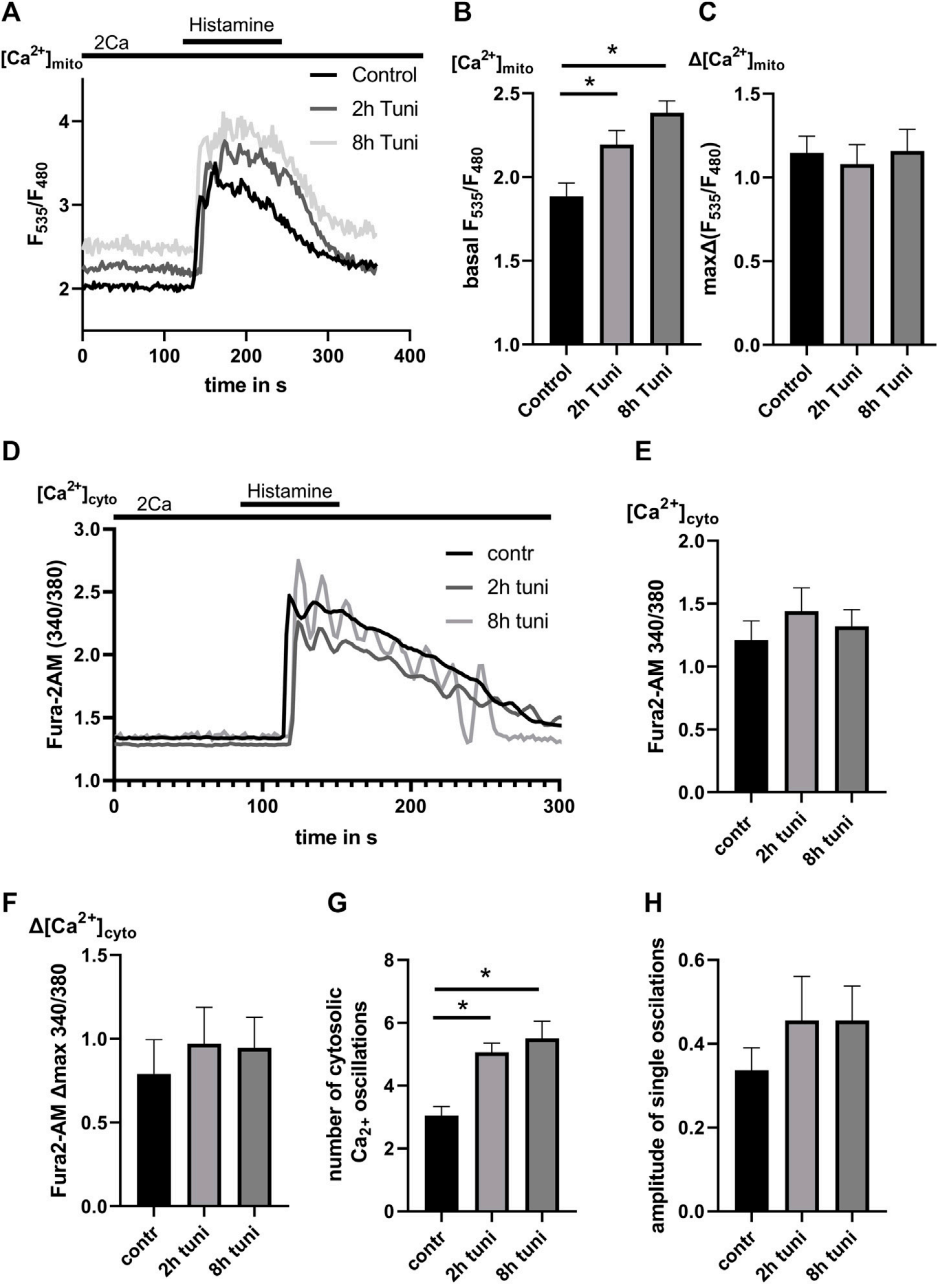
ER-stress leads to increased basal [Ca^2+^]_mito_ and IP_3_ generating agonist induced oscillations of [Ca^2+^]_cyto_. **(A)** HeLa cells stable expressing the mitochondrial targeted genetically encoded Ca^2+^ sensor 4mtd3cpv were used. Representative [Ca^2+^]_mito_ traces upon histamine induced [Ca^2+^]_ER_ release of control HeLa cells or HeLa cells treated with 0.6 µM tunicamycin for 2 h or 8 h. **(B)** Bar graphs (MEAN ± SEM) represent basal [Ca^2+^]_mito_ levels of control HeLa cells or HeLa cells treated with tunicamycin for 2 h or 8 h. **(C)** Bar graphs represent [Ca^2+^]_mito_ uptake in response to histamine treatment of control HeLa cells (*n* = 4/8/40) or HeLa cells treated with tunicamycin for 2 h (*n* = 4/8/45) or 8 h (*n* = 4/8/48). **(D)** HeLa cells labeled with the cytosolic Ca^2+^ indicator Fura-2 AM were used. Representative [Ca^2+^]_cyto_ traces upon histamine induced [Ca^2+^]_ER_ release of control HeLa cells or HeLa cells treated with tunicamycin for 2 h or 8 h. **(E)** Bar graphs represent basal [Ca^2+^]_cyto_ levels of control HeLa cells or HeLa cells treated with tunicamycin for 2 h or 8 h. **(F)** Bar graphs represent [Ca^2+^]_cyto_ uptake in response to histamine treatment of control HeLa cells or HeLa cells treated with tunicamycin for 2 h or 8 h. **(G)** Bar graphs represent the number of [Ca^2+^]_cyto_ oscillations during histamine induced [Ca^2+^]_ER_ release of control HeLa cells or HeLa cells treated with tunicamycin for 2 h or 8 h. **(H)** Bar graphs represent the amplitude of [Ca^2+^]_cyto_ oscillations during histamine induced [Ca^2+^]_ER_ release of control HeLa cells (*n* = 3/6/85) or HeLa cells treated with tunicamycin for 2 h (*n* = 3/6/98) or 8 h (*n* = 3/6/105). *p < 0.05 vs. respective control conditions carried out with one-way ANOVA with Tukey corrected posthoc test.

[Ca^2+^]_mito_ homeostasis is greatly influenced by the dynamics and quantity of structural ER-mitochondrial interaction (Patergnani et al., 2011). Accordingly, we used HeLa cells stably expressing the mitochondrial-targeted genetically encoded Ca^2+^ sensor 4mtd3cpv to investigate if changes in the stability and lifetime of MAMs affect [Ca^2+^]_mito_. We found increased basal [Ca^2+^]_mito_ levels in HeLa cells treated with tunicamycin for 2 and 8 h. In contrast, mitochondrial uptake after histamine-induced [Ca^2+^]_ER_ release was not influenced by tunicamycin induced ER-stress (Figures 3A–C). Increased basal [Ca^2+^]_mito_ changes might be the result of [Ca^2+^]_cyto_ elevations (Mukherjee et al., 2002). To exclude this possibility, we used the [Ca^2+^]_cyto_ indicator Fura-2 AM to determine basal and [Ca^2+^]_ER_ release triggered [Ca^2+^]_cyto_ levels. Neither basal [Ca^2+^]_cyto_ nor ∆[Ca^2+^]_cyto_ after [Ca^2+^]_ER_ release were affected by the treatment with tunicamycin (Figures 3D–F). Additionally, we observed an increased number of oscillations after ER Ca^2+^ release in tunicamycin treated cells (Figures 3G,H), pointing to an altered orchestration of ER and cytosolic Ca^2+^ signaling.

### ER stress leads to altered mitochondrial metabolism

**FIGURE 4:**
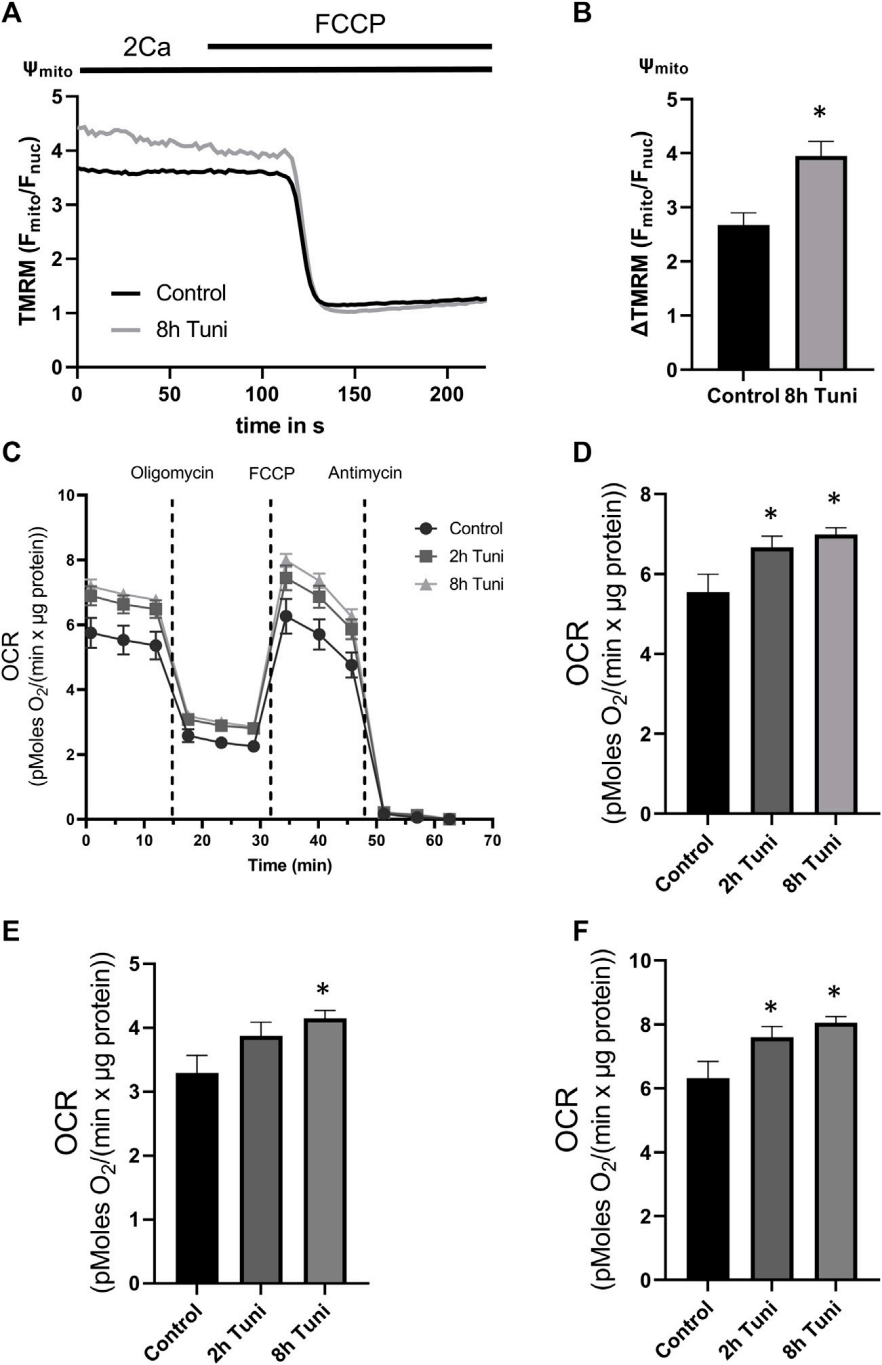
Mitochondrial membrane potential and OCR are increased in cells treated with tunicamycin. **(A)** HeLa cells labeled with the mitochondrial membrane potential dependent dye TMRM were used. Representative Ψ_mito_ (TMRM F_mito_/F_nuc_) traces upon FCCP induced loss of Ψ_mito_ of control HeLa cells or HeLa cells treated with 0.6 µM tunicamycin for 2 h or 8 h. **(B)** Bar graphs (MEAN ± SEM) show mitochondrial membrane potential normalized to fully disrupted mitochondrial membrane potential in response to FCCP of control HeLa cells (*n* = 3/6/47) or HeLa cells treated with tunicamycin for 8 h (*n* = 3/6/50). **(C)** OCR changes measured by Seahorse^®^ in control HeLa cells or HeLa cells treated with tunicamycin for 2 h or 8 h. **(D)** Bar graphs (MEAN ± SEM) show mitochondrial basal respiration of control HeLa cells or HeLa cells treated with tunicamycin for 8 h. **(E)** Bar graphs (MEAN ± SEM) show ATP-linked respiration of control HeLa cells or HeLa cells treated with tunicamycin for 8 h. **(F)** Bar graphs (MEAN ± SEM) show maximal respiratory capacity of control HeLa cells (*n* = 3/26) or HeLa cells treated with tunicamycin for 2 h (*n* = 3/21) or 8 h (*n* = 3/24). *p < 0.05 vs. respective control conditions carried out with one-way ANOVA with Tukey corrected posthoc test.

Oxidative phosphorylation (OXPHOS) is highly dependent on [Ca^2+^]_mito_ levels due to the importance of basal [Ca^2+^]_mito_ in the activity of the dehydrogenases of the tricarboxylic acid (TCA) cycle (Denton et al., 1975; Denton et al., 1978; Lawlis and Roche, 1981). Accordingly, we used tetramethylrhodamine methyl ester (TMRM) and Seahorse^®^ respirometer to analyze the effects of tunicamycin on mitochondrial membrane potential and oxygen consumption rate (OCR), respectively. We found significantly hyperpolarized mitochondria (Figures 4A,B) and increases in basal OCR, ATP-linked respiration, and maximal respiratory capacity after 8 h of tunicamycin incubation (Figures 4C–F). These data show a potential link between ER stress-induced MAM remodeling and increased mitochondrial OXPHOS *via* elevated basal [Ca^2+^]_mito_.

### Tunicamycin leads to [ATP]_ER_ coupling to oxidative [ATP]_mito_ production

**FIGURE 5:**
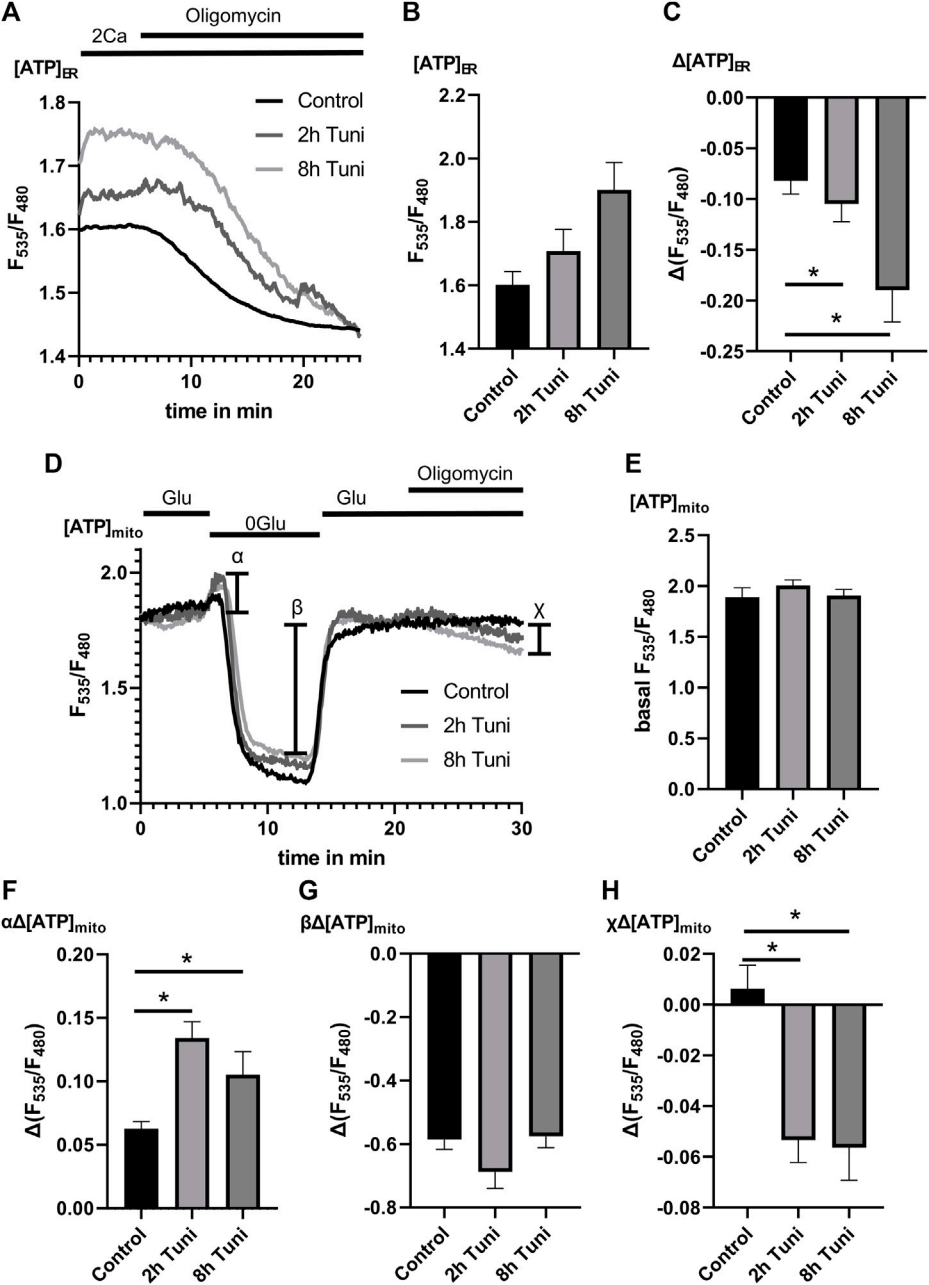
[ATP]_mito_ and [ATP]_ER_ are more dependent on oxidative metabolism under ER-stress. **(A)** HeLa cells expressing the endoplasmic reticulum targeted genetically encoded [ATP]_ER_ sensor ERAT4.01 were used. Representative [ATP]_ER_ traces upon Oligomycin challenging of control HeLa cells or HeLa cells treated with 0.6 µM tunicamycin for 2 h or 8 h. **(B)** Bar graphs (MEAN ± SEM) show basal [ATP]_ER_ of control HeLa cells or HeLa cells treated with tunicamycin for 8 h. **(C)** Bar graphs (MEAN ± SEM) show ∆[ATP]_ER_ upon challenging control HeLa cells (*n* = 4/8/31) or HeLa cells treated with tunicamycin for 2 h (*n* = 4/8/51) or 8 h (*n* = 4/8/38) with oligomycin. **(D)** HeLa cells stably expressing the mitochondrial targeted genetically encoded [ATP]_mito_ sensor mtAT1.03 were used. Representative [ATP]_mito_ traces upon consecutive glucose removal, glucose re-addition and Oligomycin addition of control HeLa cells or HeLa cells treated with tunicamycin for 2 h or 8 h. **(E)** Bar graphs (MEAN ± SEM) show basal [ATP]_mito_ of control HeLa cells or HeLa cells treated with tunicamycin for 2 h or 8 h. **(F)** Bar graphs (MEAN ± SEM) show initial ∆[ATP]_mito_ induced by glucose removal [see *α* in (D)] of control HeLa cells or HeLa cells treated with tunicamycin for 2 h or 8 h. **(G)** Bar graphs (MEAN ± SEM) show ∆[ATP]_mito_ induced by glucose removal (see β in **(D)**) of control HeLa cells or HeLa cells treated with tunicamycin for 2 h or 8 h. **(H)** Bar graphs (MEAN ± SEM) show ∆[ATP]_mito_ induced by the addition of oligomycin (see *χ* in **(D)**) of control HeLa cells (*n* = 3/6/64) or HeLa cells treated with tunicamycin for 2 h (*n* = 3/6/41) or 8 h (*n* = 3/6/37). *p < 0.05 vs. respective control conditions carried out with one-way ANOVA with Tukey corrected posthoc test.

Our data so far let us speculate that the increase in ER ATP during ER stress response (Yong et al., 2019) is achieved by a stabilization of MAMs that yield increased basal [Ca^2+^]_mito,_ and stimulated mitochondrial respiration. Accordingly, we measured the dependency of [ATP]_ER_ on oxidative mitochondrial respiration. For that purpose, we used the genetically encoded ER targeted ATP sensor ERAT4.01 (Vishnu et al., 2014) and challenged control and tunicamycin treated cells with oligomycin. We found increased basal [ATP]_ER_ and a stronger [ATP]_ER_ drop during oligomycin challenging in cells treated with tunicamycin (Figures 5A–C), pointing to an increased coupling of ER metabolism to mitochondrial oxidative phosphorylation. To further support these findings, we measured [ATP]_mito_ in mtAT1.03 stable expressing HeLa cells treated with and without tunicamycin. In our protocol, we first removed glucose, followed by glucose re-addition and challenging with oligomycin. The initial elevation of [ATP]_mito_ after glucose (Figure 5D) removal was shown to be linked to the activity of hexokinase (Depaoli et al., 2018) and cellular glycolytic activity. We could see an elevation of hexokinase activity (Figure 5F) while basal and minimum [ATP]_mito_ after glucose removal remained unchanged (Figures 5E,G), pointing to an increased aerobic glycolysis under tunicamycin treatment. The addition of oligomycin led to a reduction of [ATP]_mito_ in HeLa cells treated with tunicamycin (Figure 5H). Considering the changes of [ATP]_mito_ and [ATP]_ER_ upon tunicamycin treatment/ER stress, we conclude that increased mitochondrial oxidative phosphorylation fuels the increased availability of ATP in the ER during the early phase of ER stress.

### Knockdown of MFN2 prevents ER-stress induced stable MAMs

**FIGURE 6:**
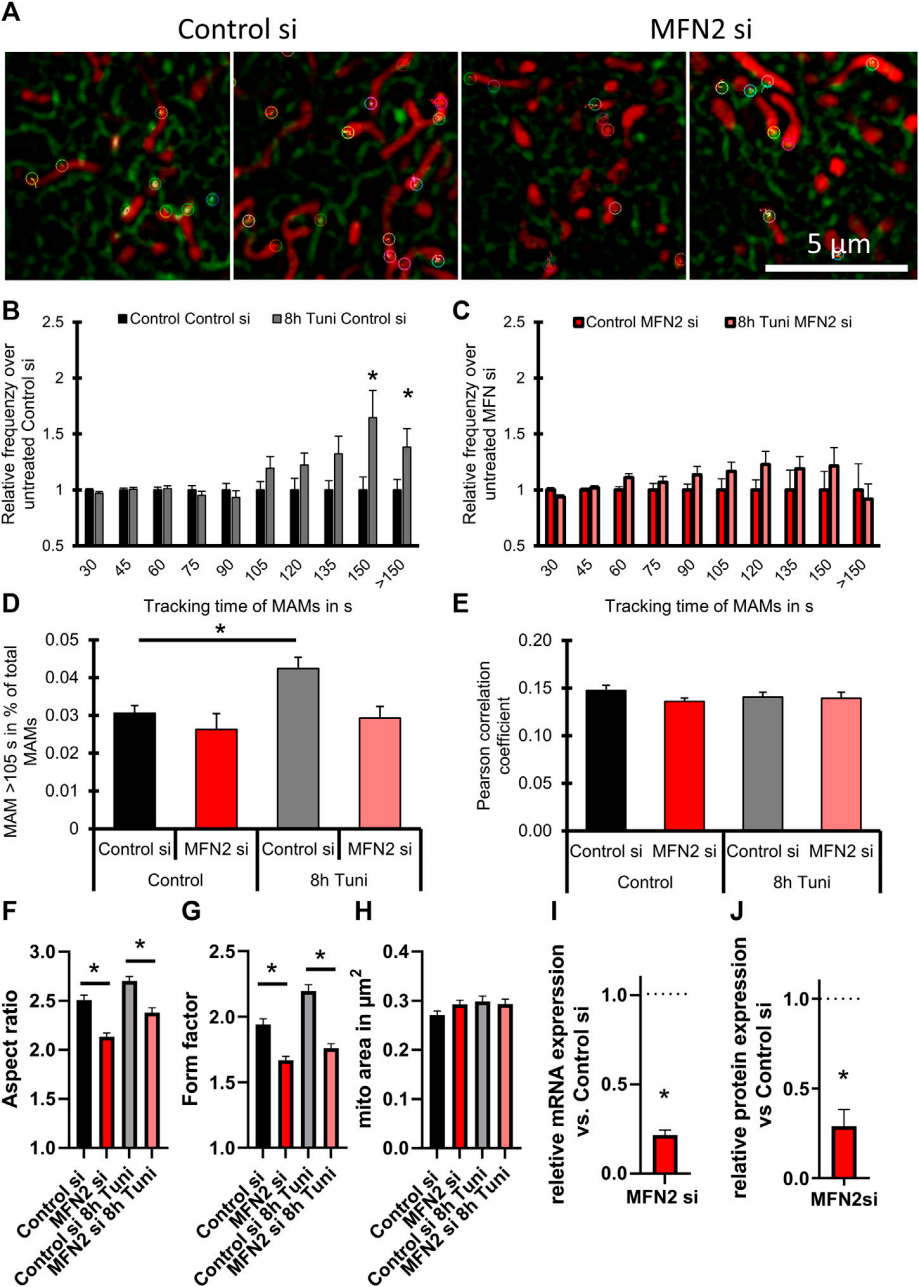
Silencing of MFN2 cancels ER-stress induced changes in MAM stability. **(A)** Representative images of HeLa cells expressing the ER marker ERAT4.01 NA, transfected with siRNA against MFN2 or Control siRNA were labeled with 100 nM TMRM and treated with 0.6 µM tunicamycin for 8 h or the respective control. Color coded markers show MAMs tracked for >105 s. **(B)** The relative frequency (MEAN ± SEM) of MAMs is plotted over the tracking time of single MAMs of cells transfected with Control siRNA and treated with tunicamycin for 8 h. **(C)** The relative frequency of MAMs (MEAN ± SEM) is plotted over the tracking time of single MAMs of cells transfected with siRNA against MFN2 and treated with tunicamycin for 8 h **(D)** A threshold of 105 s tracking time was set to define long lasting MAMs and the percentage of MAMs included in that group was plotted as a bar graph (MEAN ± SEM) for cells transfected with siRNA against MFN2 or Control siRNA and treated with tunicamycin for 8 h. **(E)** Bar graphs (MEAN ± SEM) shows Pearson’s correlation coefficient of control HeLa or HeLa cells treated with tunicamiycin for 8 h and transfected with siRNA against MFN2 or Control siRNA. **(F)** Bar graphs (MEAN ± SEM) shows mitochondrial aspect ratio of control HeLa or HeLa cells treated with tunicamiycin for 8 h and transfected with siRNA against MFN2 or Control siRNA. **(G)** Bar graphs (MEAN ± SEM) shows mitochondrial form factor of control HeLa or HeLa cells treated with tunicamycin for 8 h. **(H)** Bar graphs (MEAN ± SEM) shows mitochondrial size of control HeLa or HeLa cells treated with tunicamycin for 8 h and transfected with siRNA against MFN2 or Control siRNA (n_control si/DMSO_ = 3/35; n_control si/8h Tuni_ = 3/32; n_MFN2 si/DMSO_ = 3/34; n_MFN2 si/8h Tuni_ = 3/28). **(I)** Verification of knockdown efficiency by qRT-PCR (MEAN ± SEM) of MFN2 after treatment with control siRNA or siRNAs against MFN2 (*n* = 3). **(J)** Verification of knockdown efficiency by Western blot (MEAN ± SEM) of MFN2 after treatment with control siRNA or siRNAs against MFN2 (*n* = 3). *p < 0.05 vs. respective control conditions carried out with one-way ANOVA with Tukey corrected posthoc test.

MFN2 is known to mediate mitochondrial fission (Ngoh et al., 2012) and ER—mitochondria tethering (Naon et al., 2016; Casellas-Díaz et al., 2021). MFN2 is upregulated in response to tunicamycin induced ER stress (Ngoh et al., 2012). Knockout of MFN2 leads to increased ER stress response, including increased ER chaperone expression (Ngoh et al., 2012; Sebastián et al., 2012), ER expansion and enhancement of UPR branches (e.g., PERK, XBP-1, and ATF6) (Muñoz et al., 2013). Therefore, we investigated whether MFN2 might be involved in the formation of stable MAMs after tunicamycin treatment. We found that knockdown of MFN2 in HeLa cells prevented the formation of ER stress-induced stable MAMs (Figures 6A–D; Supplementary Figure S4; Supplementary Videos S4–S7).

Regardless, the overall proximity between ER and mitochondria calculated as Pearson correlation coefficient was not influenced by the knockdown of MFN2 (Figure 6E). The mitochondrial morphology was affected by silencing of MFN2. Aspect ratio and form factor were reduced, pointing to reduced mitochondrial interconnection, fragmentation and swelling (Figures 6F–H). Knockdown efficiency of MFN2 siRNA was measured *via* qRT-PCR and western blot and calculated to be approximately 80 and 71%, respectively (Figures 6I,J; Supplementary Figure S5).

### Silencing of MFN2 prevents the increased inter-organelle Ca^2+^ and ATP transport during ER-stress

**FIGURE 7:**
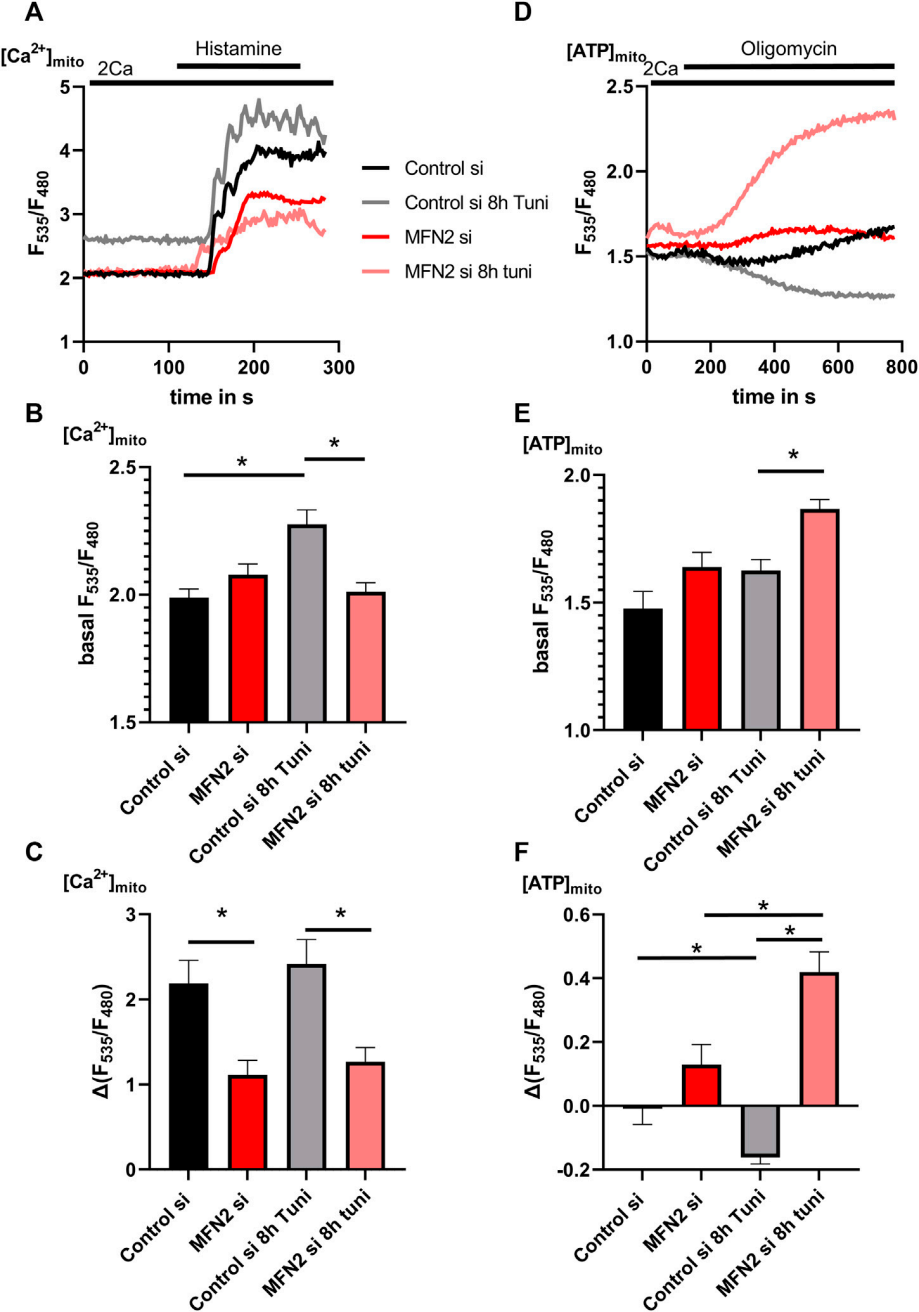
[Ca^2+^]_mito_ and [ATP]_mito_ of MFN2 silenced HeLa cells show no response on ER-stress. **(A)** HeLa cells stabile expressing the mitochondrial targeted genetically encoded Ca^2+^ sensor 4mtd3cpv were transfected with siRNA against MFN2 or Control siRNA. Representative [Ca^2+^]_mito_ traces upon histamine induced [Ca^2+^]_ER_ release of control HeLa cells or HeLa cells treated with 0.6 µM tunicamycin for 8 h. **(B)** Bar graphs (MEAN ± SEM) represent basal [Ca^2+^]_mito_ levels of control HeLa cells or HeLa cells treated with tunicamycin for 8 h. **(C)** Bar graphs (MEAN ± SEM) represent [Ca^2+^]_mito_ uptake in response to histamine treatment of control HeLa cells or HeLa cells treated with tunicamycin for 8 h (n_control si/DMSO_ = 3/9/48; n_control si/8h Tuni_ = 3/9/35; n_MFN2 si/DMSO_ = 3/9/52; n_MFN2 si/8h Tuni_ = 3/9/55) **(D)** HeLa cells stabile expressing the mitochondrial targeted genetically encoded ATP sensor mtAT1.03 were transfected with siRNA against MFN2 or Control siRNA. Representative [ATP]_mito_ traces upon oligomycin addition of control HeLa cells or HeLa cells treated with Tunicamycin for 8 h. **(E)** Bar graphs (MEAN ± SEM) represent basal [ATP]_mito_ levels of control HeLa cells or HeLa cells treated with tunicamycin for 8 h. **(F)** Bar graphs (MEAN ± SEM) represent [ATP]_mito_ upon oligomycin addition of control HeLa cells or HeLa cells treated with tunicamycin for 8 h (n_control si/DMSO_ = 3/9/89; n_control si/8h Tuni_ = 3/9/80; n_MFN2 si/DMSO_ = 3/9/66; n_MFN2 si/8h Tuni_ = 3/9/82). *p < 0.05 vs. respective control conditions carried out with one-way ANOVA with Tukey corrected posthoc test.

The neutralization of ER-stress induced stable MAMs by reduction of MFN2 expression lead to the question whether the increased exchange of Ca^2+^ and ATP between mitochondria and the ER during ER-stress is affected likewise. Silencing of MFN2 prevented the tunicamycin-induced increase of basal [Ca^2+^]_mito_ (Figures 7A,B) and led to a general reduction of [Ca^2+^]_mito_ uptake capacity during [Ca^2+^]_ER_-release (Figures 7A,C). To test the specificity of the used siRNA against MFN2 we performed a recovery experiment based on the [Ca^2+^]_mito_ uptake capacity during [Ca^2+^]_ER_-release using a mCherry-MFN2 construct which we co-transfected with or without MFN2 siRNA. Basal [Ca^2+^]_mito_ was unchanged upon knockdown or expression of mCherry-MFN2 (Supplementary Figure S6). Both, knockdown and overexpression of MFN2, lead to a reduction of [Ca^2+^]_mito_ uptake capacity, which was recovered by simultaneous transfection with the MFN2 siRNA and mCherry-MFN2 construct (Supplementary Figure S6). These data prove the specificity of the siRNA used to silence MFN2.

**FIGURE 8:**
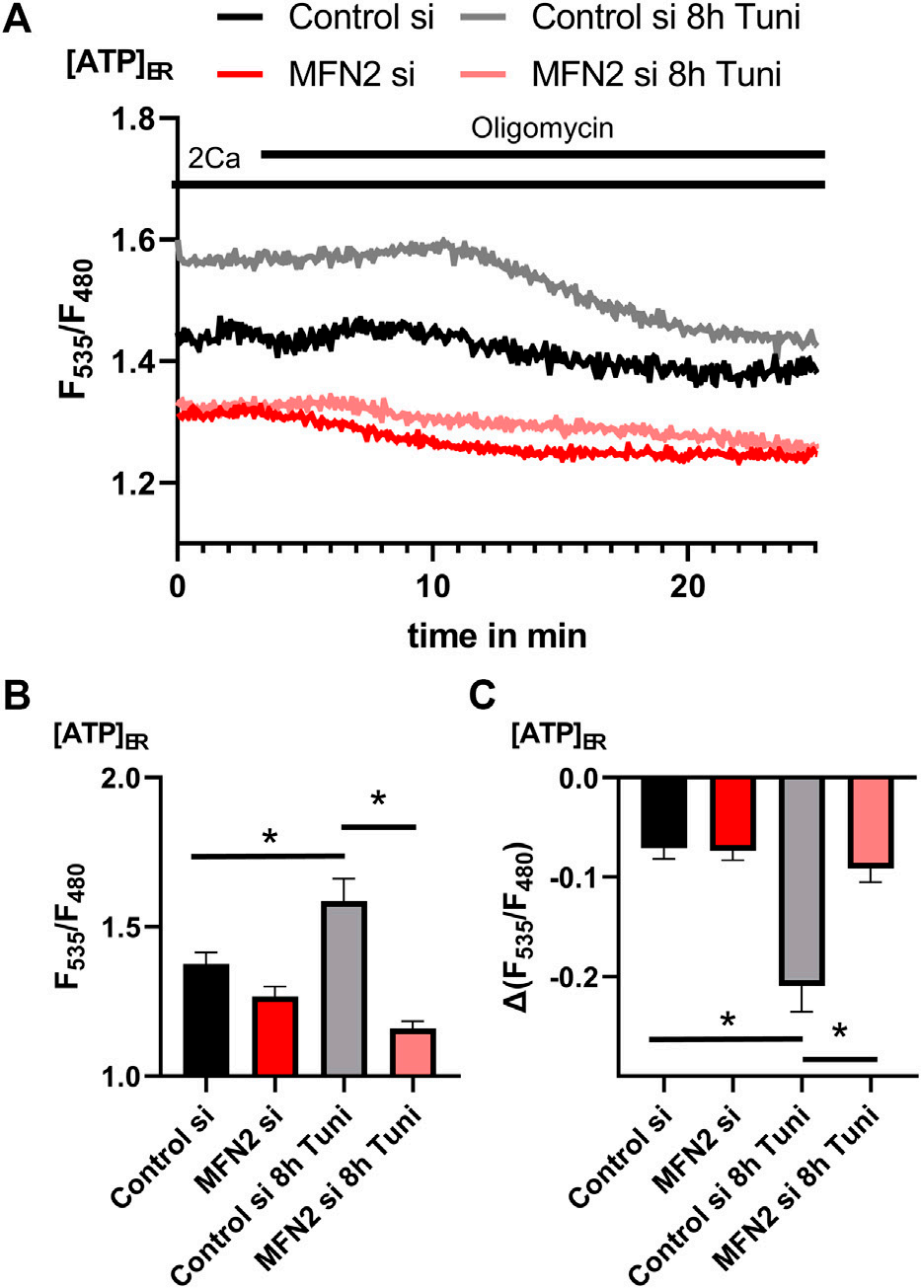
[ATP]_ER_ of MFN2 silenced HeLa cells show no response on ER-stress. **(A)** HeLa cells transfected with siRNA against MFN2 or Control siRNA and expressing the endoplasmic reticulum targeted genetically encoded [ATP]_ER_ sensor ERAT4.01 were used. Representative [ATP]_ER_ traces upon oligomycin challenging of control HeLa cells or HeLa cells treated with 0.6 µM tunicamycin for 8 h. **(B)** Bar graphs (MEAN ± SEM) show basal [ATP]_ER_ of control HeLa cells or HeLa cells treated with tunicamycin for 8 h transfected with siRNA against MFN2 or Control siRNA. **(C)** Bar graphs (MEAN ± SEM) show ∆[ATP]_ER_ upon challenging control HeLa cells or HeLa cells treated with tunicamycin for 2 h transfected with siRNA against MFN2 or Control siRNA or 8 h with oligomycin (n_control si/DMSO_ = 3/8/27; n_control si/8h Tuni_ = 3/8/30; n_MFN2 si/DMSO_ = 3/8/35; n_MFN2 si/8h Tuni_ = 3/8/38). *p < 0.05 vs. respective control conditions carried out with one-way ANOVA with Tukey corrected posthoc test.

Measurements of [ATP]_mito_ revealed increased basal ATP concentrations (Figures 7D,E) and a substantial increase in mitochondrial ATP in MFN2 depleted cells upon oligomycin (Figures 7D,F). These data indicate that MFN2 silencing caused a switch of the F_1_F_O_ATPase into the reverse mode, consuming ATP to maintain the mitochondrial membrane potential. Since ER ATP is coupled to these events, we observed increased basal [ATP]_ER_ (Figures 8A,B) and a strong drop of [ATP]_ER_ during the perfusion with oligomycin in cells treated with tunicamycin (Figures 8A,C), as shown before (Figures 5A–C). Knockdown of MFN2 impedes that dynamic and negates the effect of tunicamycin on ER ATP in HeLa cells (Figures 8A–C), pointing to an inability of the ER to utilize ATP from mitochondria upon the induction of ER stress.

## Discussion

While the transport of ATP between mitochondria and the cytosol is well described a long time (Henderson and Lardy, 1970; Chevrollier et al., 2011), the transport mechanisms for ATP into the ER have been recently discovered (Klein et al., 2018; Yong et al., 2019). Hence, interorgenellar Ca^2+^ fluxes between the ER and mitochondria through MAMs are well established (Rizzuto et al., 1998; Giacomello et al., 2010). We used structured illumination super-resolution microscopy to analyze if ATP channeling from the mitochondria to the ER is bound to MAMs. For that approach, we analyzed the lifetime of MAMs in HeLa cells and correlated these results with live cell measurements of an array of genetically encoded sensors for ATP and Ca^2+^ to investigate the crosstalk between the ER and mitochondria under ER stress conditions.

In the present work, we describe that ER stress induces a longer lifetime of MAMs while no change in ER-mitochondrial co-localization or mitochondrial morphology was observed. Previous studies showed that tunicamycin increases tight junctions between mitochondria and the ER using electron microscopy (Csordás et al., 2006; Bravo et al., 2011). We speculate that the tightness of MAMs is directly correlated to their lifetime. Anyhow, fluorescence microscopy results of the same study (Bravo et al., 2011) using the Manders correlation coefficient showed increased co-localization between the ER and mitochondria, which we could not confirm. The binary nature of the Manders correlation coefficient paired with insufficient resolution of confocal microscopy and the reported clustering of ER and mitochondria in the cellular center display potential pitfalls. Structured illumination microscopy delivers high resolution, necessary to detect MAMs, and the Pearson correlation coefficient is better suited to investigate organelle interaction because no thresholding of fluorescence data is nessesary. The unaltered general proximity between ER and mitochondria during ER stress found in our study shows the importance of MAM lifetime measurements to quantify the quality of MAMs besides their amount and to interpret the Ca^2+^ and ATP dynamics during ER stress. Increased basal mitochondrial Ca^2+^ levels during ER stress might be the result of a combination of an increased ER Ca^2+^ leak (Koshenov et al., 2021) and more stable MAMs, both potentially causing mitochondrial Ca^2+^ overload and apoptosis at a later stage of ER stress (Deniaud et al., 2008). Hence, we observed that initially the ER stress-based increased basal mitochondrial Ca^2+^ led to increased mitochondrial membrane potential and OXPHOS due to the activation of the TCA-cycle (Denton et al., 1975; Denton et al., 1978; Lawlis and Roche, 1981). Basal mitochondrial ATP levels remained unchanged upon tunicamycin treatment. Anyhow, we detected an increased drop in [ATP]_mito_ after addition of oligomycin in tunicamycin treated cells. This indicates a higher metabolic flux of ATP most probably towards the ER canceling out increased mitochondrial ATP production.

As shown elsewhere (Yong et al., 2019), tunicamycin treatment causes increased oxidative mitochondrial metabolism, which directly translates to the ER ATP levels. While cytosolic Ca^2+^ concentrations remained stable and IP_3_-induced ER Ca^2+^ release remained unchanged during early phases of ER stress, the number of Ca^2+^ oscillations was increased during ER stress, pointing to increased interaction between ER and mitochondria. Notably, because Ca^2+^-antagonized ATP transport into the ER (Yong et al., 2019) ER ATP might be related with the integrity of MAMs. Hence, increased cytosolic Ca^2+^ activates the mitochondrial fission protein dynamin-related protein 1 (DRP1) (Han et al., 2008) which is localized to MAMs (Zhang et al., 2018). Disruption of MAMs impedes ATP transfer from the mitochondria to the ER and thus would explain the effects of cytosolic Ca^2+^ changes on ER ATP levels. Whether or not the ER ATP/ADP exchanger AXER (Klein et al., 2018) is located to MAMs or another transporter is involved in the ATP uptake remains elusive.

In the present work we demonstrate that the MAM protein MFN2 is involved in the formation of MAMs with a long lifetime during ER stress. The function of MFN2 in the orchestration of MAMs is highly debated and the protein is described as ER—mitochondria tether (de Brito and Scorrano, 2008; Naon et al., 2016) but also as spacer ensuring a certain distance between the organelles (Filadi et al., 2015). Our findings confirm these reports, as we found decreased mitochondrial Ca^2+^ uptake originating from ER Ca^2+^ release in cells silenced for MFN2 or overexpressing mCherry-MFN2. This shows that the tether distance modulation by MFN2 is highly important for Ca^2+^ exchange. In the present work, MFN2 knockdown did not change MAM lifetime or ER-mitochondrial proximity measured with Pearson correlation coefficient under control conditions. However, MFN2 knockdown prevented increased MAM lifetimes during ER stress, pointing to a tethering function of MFN2 during ER stress. In line with these findings, MFN2 expression was reported to be increased during tunicamycin-induced ER stress (Ngoh et al., 2012). Hence, ER stress upregulates the expression of GR78 (Chen et al., 2014) and HRD1 (Kaneko et al., 2002), which are capable to degrade PINK (Guardia-Laguarta et al., 2019), which phosphorylates MFN2. Subsequent ubiquitation of MFN2 by PARKIN marks MFN2 for proteasomal degradation (Chen and Dorn, 2013). Thus, ER-stress might downregulate the proteasomal degradation of MFN2.

Since ER and mitochondrial proximity were not changed by knockdown of MFN2, the tethering of ER and mitochondria mediated by MFN2 likely is specific to a sub-fraction of contact sites between both organelles. Considering our mitochondrial Ca^2+^ measurements, we assume that this MAM sub-fraction is important for both basal Ca^2+^ concentration and mitochondrial Ca^2+^ uptake capacity during ER-Ca^2+^ release. Further, a clear reversal of the F_1_F_O_ATPase (Depaoli et al., 2018) was observed in cells silenced for MFN2, most probably as a result of higher glycolytic metabolism and to protect the mitochondrial membrane potential from collapse. This mechanism was amplified by ER-stress. We also observed a fragmentation and swelling of mitochondria, which is generally associated with an anaerobic glycolytic state of cell metabolism (Westermann, 2012). As basal mitochondrial Ca^2+^ was not increased in MFN2 silenced cells, a Ca^2+^-overload dependent mitochondrial swelling (Giorgi et al., 2012) can be excluded. A clear directional relation between mitochondrial morphology and metabolism cannot be drawn. The metabolic switch caused by ER-stress in control cells was prevented or even turned into the opposite by knockdown of MFN2. This is evident regarding the basal [ATP]_ER_ and oligomycin induced loss of [ATP]_ER_ pointing to a OXPHOS driven ATP supply of the ER during ER-stress.

To conclude, our results show that ER-stress induced increases in [ATP]_ER_ result from changes in MAM dynamics, causing a shift of the cellular metabolic state towards OXPHOS driven ATP production. We hypothesize, that the ATP transport into the ER lumen as well as ER-Ca^2+^ channeling to the mitochondria is mediated through specialized MAMs, which are dependent on the expression of MFN2.

## Data Availability

The raw data supporting the conclusion of this article will be made available by the authors, without undue reservation.
