# Supplementary material for: MFN2 mediates ER-mitochondrial coupling during ER stress through specialized stable contact sites
Source: Front Cell Dev Biol. 2022 Sep 8;10:918691. doi: 10.3389/fcell.2022.918691 (PMC9493370; doi:10.3389/fcell.2022.918691)
Supplement: Supplementary file 2 [file DataSheet1.PDF]

Supplementary figure 1

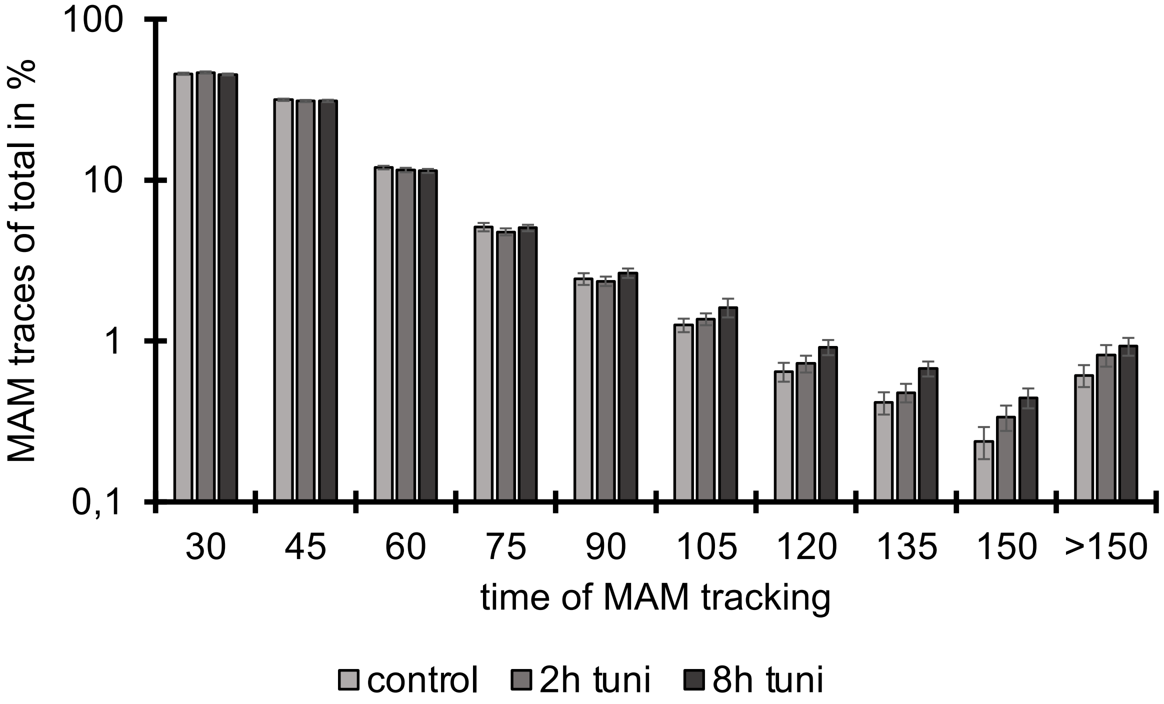


Supplementary figure 1: Treatment with tunicamycin increases MAM tracking duration in HeLa cells. HeLa cells expressing the ER marker ERAT4.01 NA were labeled with 100 nM TMRM and treated with 0.6 µM tunicamycin for 2 or 8 hours or the respective DMSO control. The frequency of MAMs is plotted over the tracking time of single MAMs n = 4/32 (days/cells).

Supplementary figure 2

A

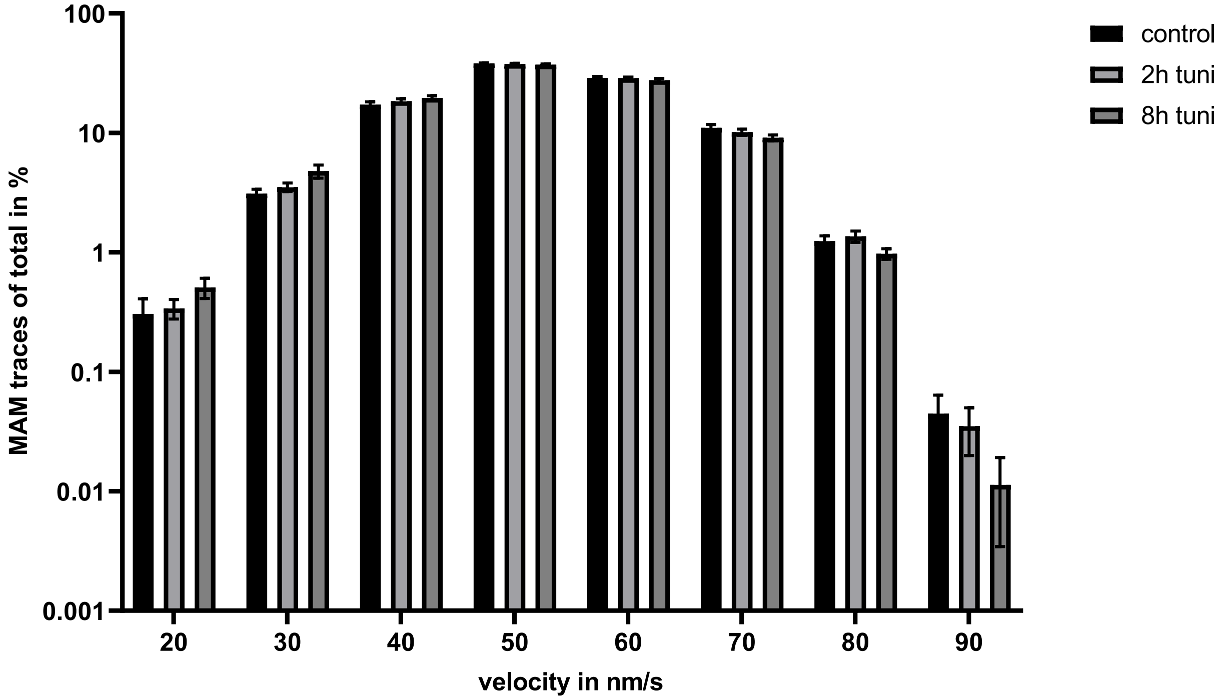


B

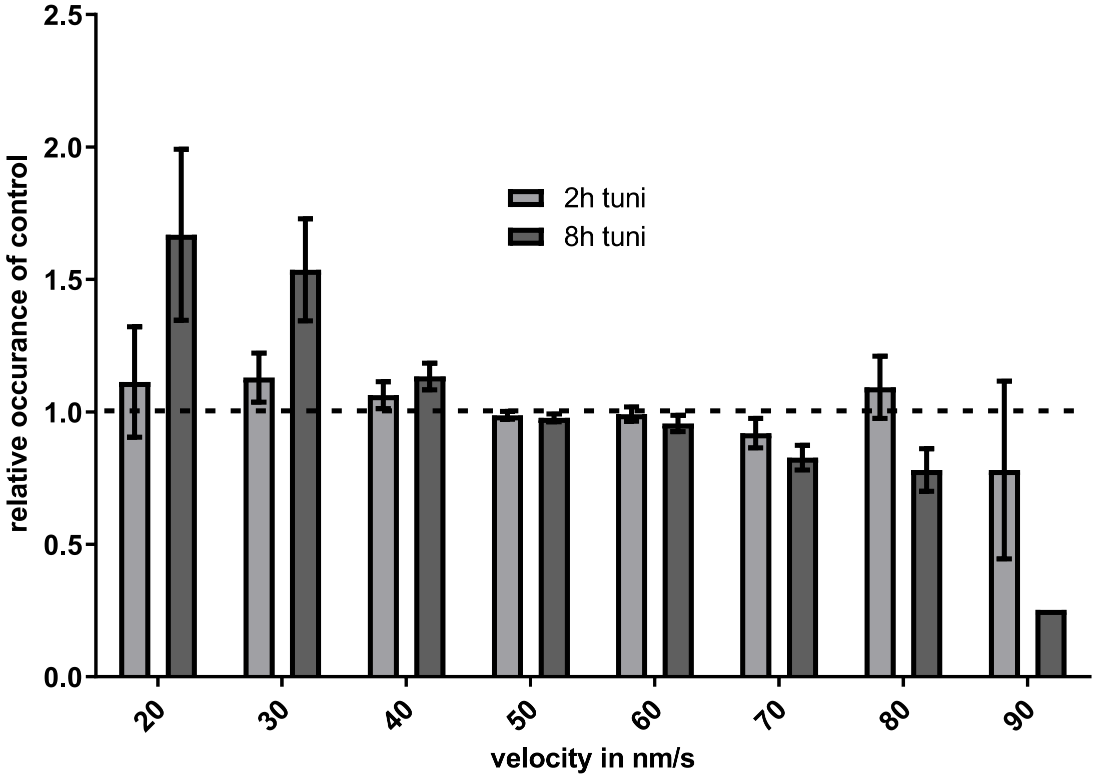


Supplementary figure 2: Treatment with tunicamycin decreases MAM tracking velocity in HeLa cells. (A) HeLa cells expressing the ER marker ERAT4.01 NA were labeled with 100 nM TMRM and treated with 0.6 μM tunicamycin for 2 or 8 hours or the respective DMSO control. The frequency of MAMs is plotted over the traveling velocity of single MAMs. (B) HeLa cells expressing the ER marker ERAT4.01 NA were labeled with 100 nM TMRM and treated with 0.6 μM tunicamycin for 2 or 8 hours or the respective DMSO control. The relative frequency of MAMs is plotted over the traveling velocity of single MAMs n = 4/32 (days/cells).

# Supplementary figure 3

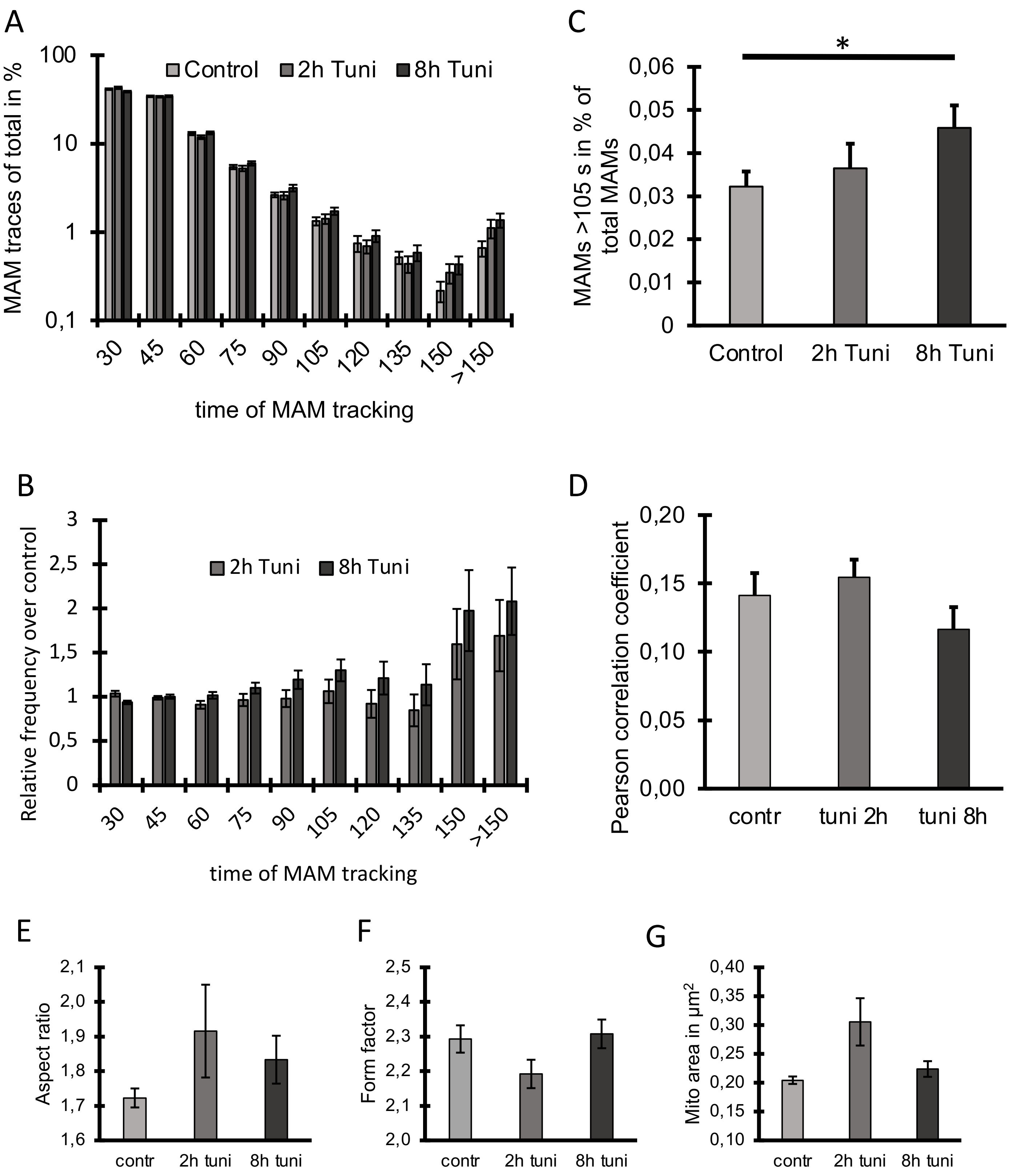

Supplementary figure 3: Treatment with tunicamycin increases MAM tracking duration in EaHy cells. EaHy cells expressing the ER marker ERAT4.01 NA were labeled with 100 nM TMRM and treated with 0.6 µM tunicamycin for 2 or 8 hours or the respective DMSO control. The frequency (A) and relative frequency (B) of MAMs is plotted over the tracking time of single MAMs n = 4/32 (days/cells). (C) A threshold of 105 s tracking time was set to define long lasting MAMs and the percentage of MAMs included in that group was plotted as a bar graph (MEAN±SEM) for cells treated with tunicamycin for 2 or 8 hours or the respective DMSO control. (D) Bar graphs (MEAN±SEM) shows Pearson's correlation coefficient of control EaHy or EaHy cells treated with tunicamycin for 2 h or 8 h. (E) Bar graphs (MEAN±SEM) shows mitochondrial aspect ratio of control HeLa or EaHy cells treated with tunicamycin for 2 h or 8 h. (F) Bar graphs (MEAN±SEM) shows mitochondrial form factor of control EaHy or EaHy cells treated with tunicamycin for 2 h or 8 h. (G) Bar graphs (MEAN±SEM) shows mitochondrial size of control EaHy or EaHy cells treated with tunicamycin for 2 h or 8 h. n = 4/32 (days/cells) * $p < 0.05$ 8h Tuni vs. respective control conditions carried out with one-way ANOVA with Tukey corrected posthoc test.

# Supplementary figure 4

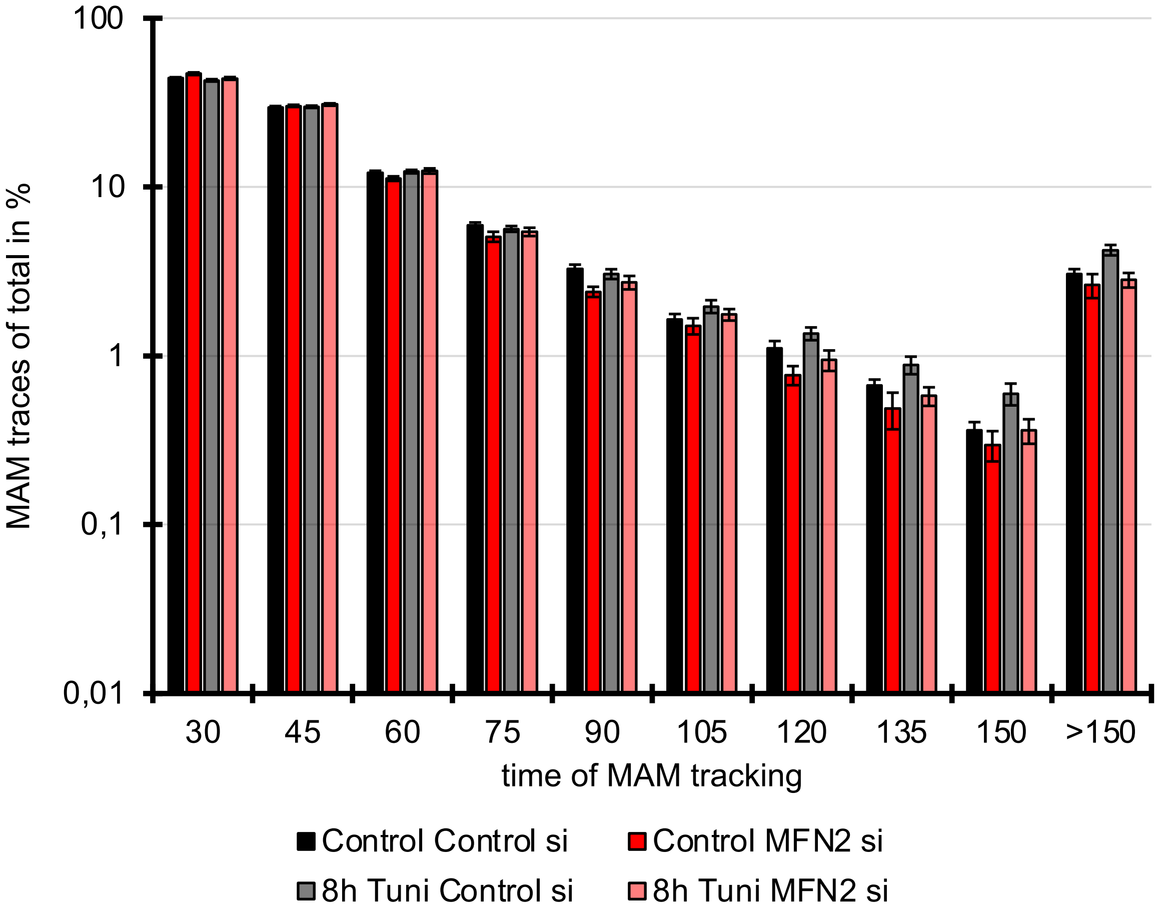


Supplementary figure 4: Knockdown of MFN2 inhibits the effect of tunicamycin on MAM tracking duration but not MAM traveling velocity.The frequency (MEAN±SEM) of MAMs is plotted over the tracking time of single MAMs of cells transfected with Control siRNA or MFN2 siRNA and treated with tunicamycin for 8 hours. n = 4/32 (days/cells)

Supplementary figure 5

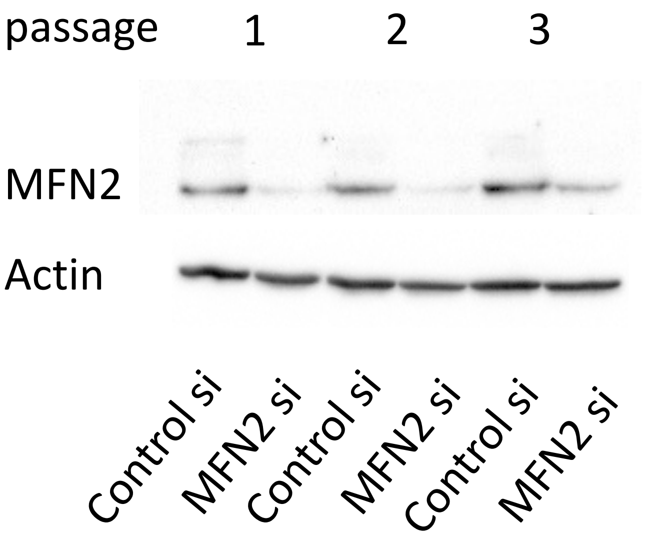


Supplementary figure 5: Quantification of knockdown efficiency of MFN2 by Western blot. Verification of knockdown efficiency of MFN2 after treatment with scrambled control siRNA or siRNAs against MFN2. For Western blot Actin was used as a loading control.

Supplementary figure 6

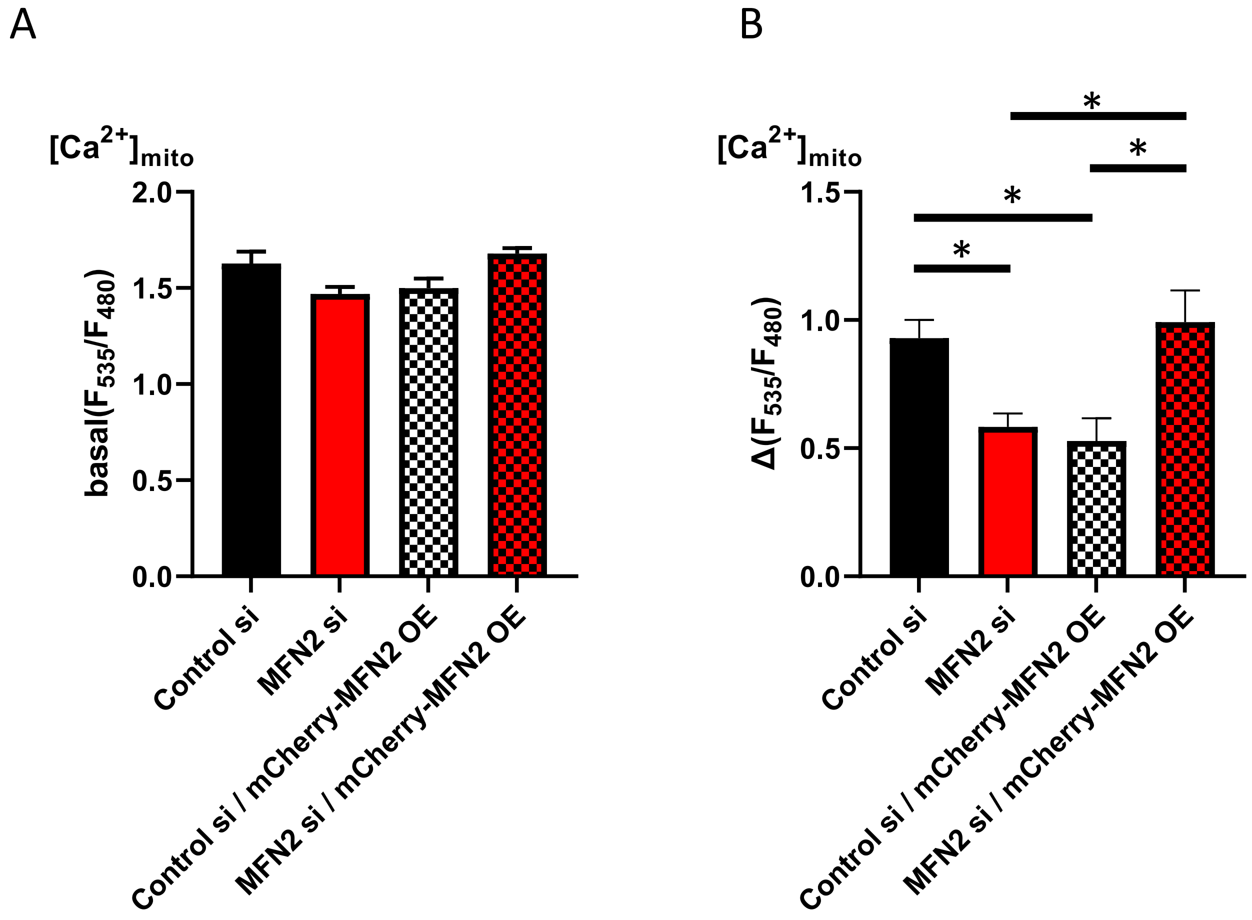


Supplementary figure 6: Overexpression of mCherry-MFN2 recovers the phenotyp of HeLa cell depleted form MFN2 using siRNA. (A) Bar graphs (MEAN±SEM) represent basal $[Ca^{2+}]_{mito}$ of control HeLa cells or HeLa cells overexpressing mCherry-MFN2 and/or were treated with siRNA against MFN2. Bar graphs (MEAN±SEM) represent $[Ca^{2+}]_{mito}$ uptake in response to histamine treatment of control HeLa cells or HeLa cells overexpressing mCherry-MFN2 and/or were treated with siRNA against MFN2. ($n_{Control\ si}$ = 3/8/70; $n_{MFN2\ si}$= 3/8/96; $n_{Control\ si/mCherry\text{-}MFN2}$ = 3/8/24; $n_{MFN2\ si/mCherry\text{-}MFN2}$= 3/8/25). * $p < 0.05$ carried out with one-way ANOVA with Tukey corrected posthoc test.
